# Different alternations of static and dynamic brain regional topological metrics in schizophrenia and obsessive-compulsive disorder

**DOI:** 10.1192/j.eurpsy.2021.1397

**Published:** 2021-08-13

**Authors:** L. Luo, Y. Yang, Q. Gong, F. Li

**Affiliations:** 1 Huaxi Mr Research Centre (hmrrc), Department Of Radiology, West China Hospital of Sichuan University, Sichuan, China; 2 Department Of Psychiatry, West China Hospital of Sichuan University, Chengdu, China; 3 Huaxi Mr Research Centre (hmrrc), Department Of Radiology, West China Hospital of Sichuan University, Chengdu, China

**Keywords:** graph theory, schizophrénia, Obsessive-Compulsive disorder, dynamic functional connectivity

## Abstract

**Introduction:**

Though schizophrenia (SZ) and obsessive-compulsive disorder (OCD) are conceptualized as distinct clinical entities, they do have notable symptom overlap and a tight association. Graph-theoretical analysis of the brain connectome provides more indicators to describe the functional organization of the brain, which may help us understand the shared and disorder-specific neural basis of the two disorders.

**Objectives:**

To explore the static and dynamic topological organization of OCD and SZ as well as the relationship between topological metrics and clinical variables.

**Methods:**

Resting state functional magnetic resonance imaging data of 31 OCD patients, 49 SZ patients, and 45 healthy controls (HC) were involved in this study (Table 1). Using independent component analysis to obtain independent components (ICs) (Figure 1), which were defined as nodes for static and dynamic topological analysis.

**Figure:**
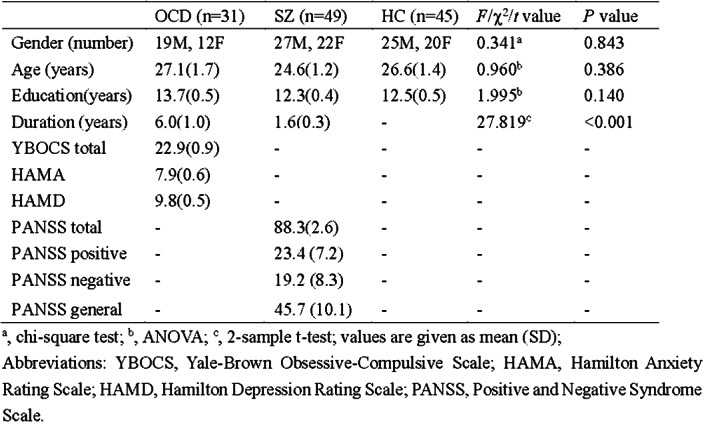

**Figure:**
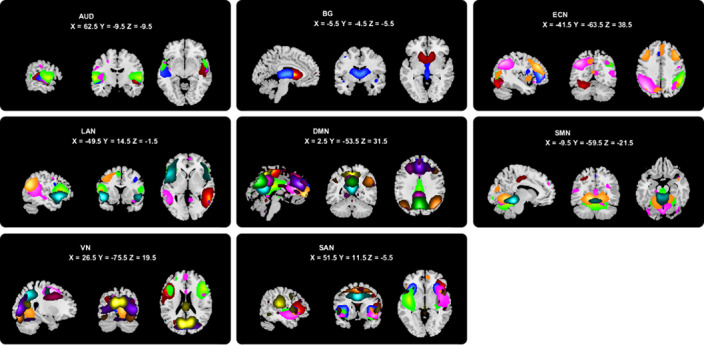

**Results:**

Static analysis showed the global efficiency of SZ was higher than HC. For nodal degree centrality, OCD exhibited decreased degree centrality in IC59 (located in visiual network) (P = 0.03) and increased degree centrality in IC38 (located in salience network) (P = 0.002) compared with HC. Dynamic analysis showed OCD exhibited decreased dynamics of degree centrality in IC38 (P = 0.003) compared with HC, which showed a negative correlation with clinical scores in OCD. While SZ showed decreased dynamics of degree centrality in IC76 (located in sensory motor network) compared with OCD (P=0.009), which showed a positive correlation with clinical scores in SZ (Figure 2).

**Figure:**
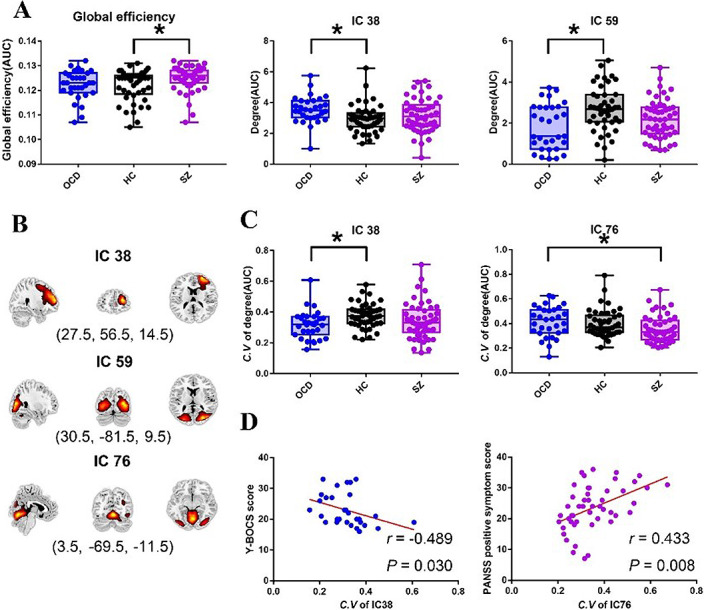

**Conclusions:**

These changes are suggestive of disorder-specific alternation of static and dynamic brain topological organization in OCD and SZ.

